# Supplementary material for: Signal Quality Analysis for Long-Term ECG Monitoring Using a Health Patch in Cardiac Patients
Source: Sensors (Basel). 2023 Feb 14;23(4):2130. doi: 10.3390/s23042130 (PMC9965306; doi:10.3390/s23042130)
Supplement: Supplementary file 1 [file sensors-23-02130-s001.zip › Supplementary Files.pdf]

# Supplementary Material

All the patients annotated their activities in a diary during the study. The annotated activities can be seen in Table S1.

**Table S1.** Questionnaires from the patients during the 5 days recording

| *Patient* | *Activity* | *Duration (min)* | *Day* | *Patient* | *Activity* | *Duration (min)* | *Day* | *Patient* | *Activity* | *Duration (min)* | *Day* |
|---|---|---|---|---|---|---|---|---|---|---|---|
| 1 | Exercise stress test | 10 | 1 | 4 | Exercise stress test | 15 | 1 | 6 | Exercise stress test | 15 | 1 |
| 1 | Walking | 60 | 3 | 4 | Cycling | 35 | 2 | 6 | Cycling | 52 | 2 |
| 1 | Walking | 60 | 3 | 4 | Walking | 60 | 2 | 6 | Cycling | 29 | 2 |
| 1 | Walking | 45 | 4 | 4 | Walking | 60 | 2 | 6 | Cycling | 174 | 3 |
| 2 | Exercise stress test | 10 | 1 | 4 | Walking | 60 | 3 | 6 | Cycling | 140 | 4 |
| 2 | Walking | 60 | 2 | 4 | Walking | 60 | 3 | 6 | Cycling | 240 | 6 |
| 2 | Cycling | 60 | 3 | 4 | Walking | 60 | 3 | | | | |
| 2 | Walking | 60 | 4 | 4 | Walking | 60 | 4 | | | | |
| 2 | Walking with dog | 60 | 5 | 4 | Walking | 60 | 4 | | | | |
| 2 | Walking | 60 | 6 | 4 | Walking | 60 | 5 | | | | |
| 3 | Exercise stress test | 10 | 1 | 5 | Exercise stress test | 16 | 1 | | | | |
| 3 | Walking | 60 | 1 | 5 | Cycling | 40 | 1 | | | | |
| 3 | Working in the garden | 480 | 2 | 5 | Fitness | 60 | 2 | | | | |
| 3 | Working in the garden | 400 | 3 | 5 | Climbing stairs (150 steps) | 5 | 5 | | | | |
| 3 | Walking | 120 | 4 | 5 | Fitness | 60 | 6 | | | | |

Figure S1 includes the $SQI_{avg}$ results with the 6 patients. It is observed that the data remains stable in the interval between 0.7-0.8, which is an indicator of the good ECG coming from the vital signs patch. However, it is not included in the analyses because this SQI is the worst-performing in the QDB classification.

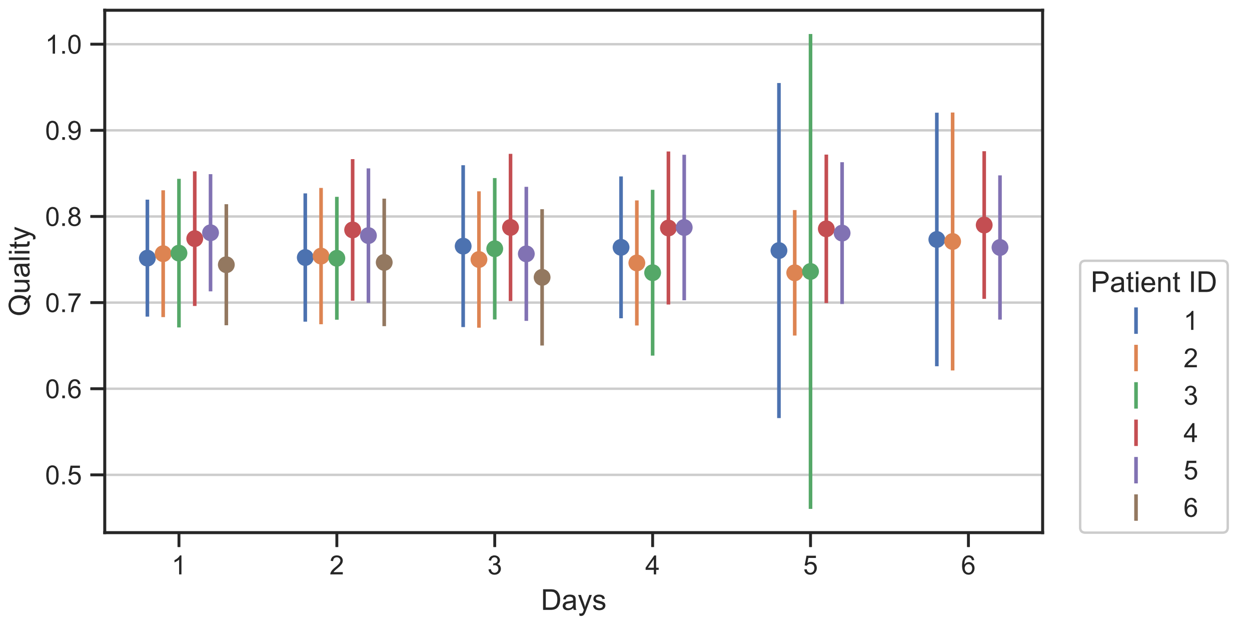


**Figure S1.** Data quality over time for all patients with $SQI_{avg}$ .
